# Artificial intelligence in tumor subregion analysis based on medical imaging: A review

**DOI:** 10.1002/acm2.13321

**Published:** 2021-06-24

**Authors:** Mingquan Lin, Jacob F. Wynne, Boran Zhou, Tonghe Wang, Yang Lei, Walter J. Curran, Tian Liu, Xiaofeng Yang

**Affiliations:** ^1^ Department of Radiation Oncology and Winship Cancer Institute Emory University Atlanta Georgia USA

**Keywords:** tumor subregion analysis, artificial intelligence, deep learning, medical imaging, medical image analysis

## Abstract

Medical imaging is widely used in the diagnosis and treatment of cancer, and artificial intelligence (AI) has achieved tremendous success in medical image analysis. This paper reviews AI‐based tumor subregion analysis in medical imaging. We summarize the latest AI‐based methods for tumor subregion analysis and their applications. Specifically, we categorize the AI‐based methods by training strategy: supervised and unsupervised. A detailed review of each category is presented, highlighting important contributions and achievements. Specific challenges and potential applications of AI in tumor subregion analysis are discussed.

## 1 INTRODUCTION

In current research and clinical practice, solid tumors are usually assumed to be homogeneous (or heterogeneous with similar distribution) throughout their volumes.^1^, ^2^, ^3^, ^4^, ^5^ However, recent studies have shown that, for some histologies, discrete tumor regions may be more biologically aggressive than others and may play a dominant role in disease progression.^6^, ^7^, ^8^ Neglecting such tumor heterogeneity at various spatial and temporal scales can lead to failures in prognosis and treatment.^6^ Medical imaging has been shown to be able to reveal and quantify the heterogeneity within tumors.^9^, ^10^, ^11^ Individual tumors can then be divided into subregions based on detected regional variations. Diagnosis, prognosis, and evaluation of treatment response can be performed individually within these subregions and have proved superior to a simple analysis of the whole tumor.^12^, ^13^ Tumor subregions may also be utilized in imaging‐based “dose painting,” delivering a specific dose to a subregion target to provide better treatment outcomes.^14^, ^15^ Therefore, accurate detection and analysis of tumor subregions are of great clinical and research interest.

Over the last few years, artificial intelligence (AI) has been applied wtih tremendous success in the field of medical imaging.^16^, ^17^, ^18^, ^19^, ^20^, ^21^, ^22^, ^23^, ^24^ Many AI‐based methods have been proposed to locate and analyze tumor subregions for a variety of imaging modalities and clinical tasks. In this study, we review the applications of supervised and unsupervised AI models in imaging‐based tumor subregion analysis. With this survey, we aim to summarize the latest developments of AI applications in imaging‐based tumor subregion analysis and highlight contributions, identify challenges, and outline future trends.

## 2 ARTIFICIAL INTELLIGENCE

AI is a field that seeks to enable machines to learn from experience, think like humans, and perform human‐like tasks. Machine learning (ML) is a discipline within AI, in which computers are trained to automatically improve performance on specific tasks based on experience. Training methods in ML are broadly composed of supervised, semi‐supervised, or unsupervised strategies, each with decreasing need for human input. Within ML, deep learning (DL) employs multilayer (“deep”) networks of mathematical functions initially intended to imitate the structure and function of the human brain, thereby fundamentally creating a mapping from one representational domain to another (e.g., categorizing photos to names of the objects they contain). Both supervised and unsupervised methods are commonly used in DL for medical image analysis.

## 3 LITERATURE SEARCH

**Fig. 1:**
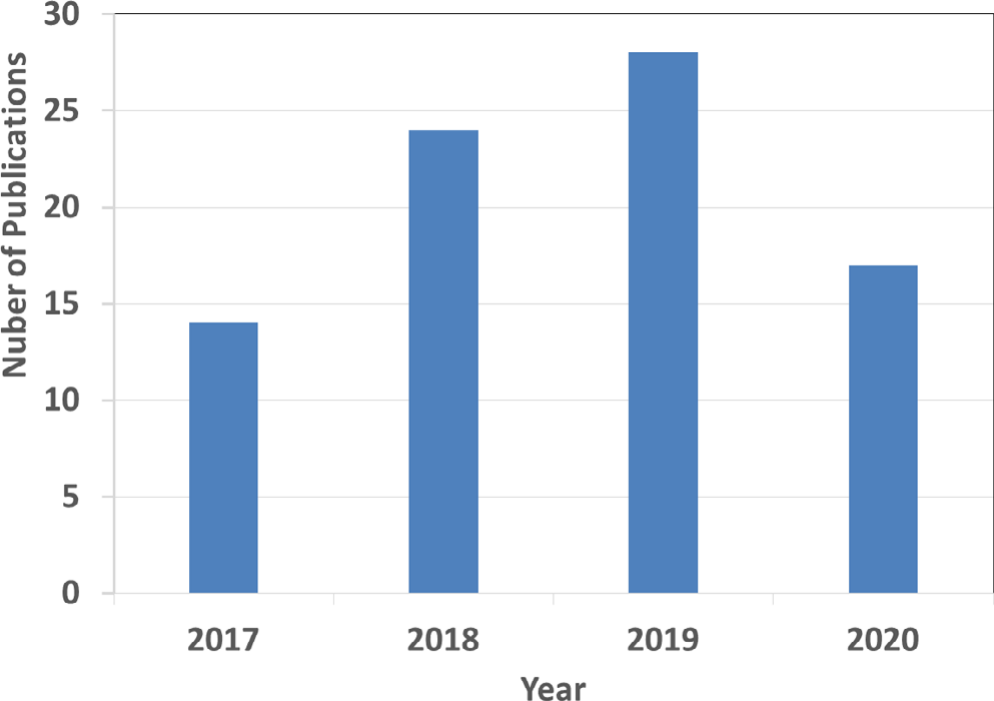
Number of publications in AI‐based tumor subregion analysis. “2020” only covers the first five months of 2020

The scope of this review is limited to the applications of AI in tumor subregion analysis. Peer‐reviewed journal publications appearing between January 1, 2017, and May 30, 2020, were collected from various databases including Google Scholar, PubMed, and Web of Science. The search was conducted by keyword, including machine learning, deep learning, intratumor, subregion, subvolume, voxel‐based, overall survival, and clustering. Publications describing the methods of the top three performers in the Brain tumor segmentation (BraTS) challenge from 2017 to 2019 were included. For all other body sites, the included publications are listed in tables accompanying each dedicated section. A total of 88 papers were identified discussing AI applications in imaging‐based tumor subregion analysis. The number of publications is plotted by year in Fig. 1.

## 4 AI IN TUMOR SUBREGION ANALYSIS OF MEDICAL IMAGES

**Fig. 2:**
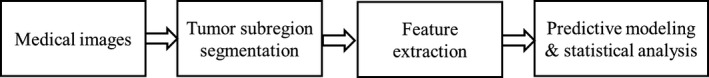
Workflow of a general AI in tumor subregion analysis of medical images

Figure 2 shows a general workflow of AI in tumor subregion analysis of medical images.

### 4.A Supervised learning in tumor subregion analysis of medical images

In supervised learning, an algorithm is trained to approximate a hypothetical function $f\left(\cdot \right)$ which maps an input (x) to an output (Y), that is, $Y=f\left(x\right)$ without error. The goal is to formulate a reasonable approximation of this function so that the output ($Y^{\prime }$) that would result from new inputs ($x^{\prime }$) can be accurately predicted. The least‐absolute‐shrinkage‐and‐selection‐operator (LASSO), random forest (RF), support vector machine (SVM), and artificial neural network (ANN) are algorithms widely used for this task. The Lasso is a shrinkage and feature selection method for linear regression^25^ which minimizes the sum of squared errors and the sum of the absolute value of coefficients. RF is an ensemble learning algorithm that boosts performance by combining the results of many weaker algorithms, effectively reducing overfitting and building a model that is robust for discrete values in the feature space.^26^ The objective of the SVM algorithm is to find a decision boundary that maximizes the separation of different classes of data in the feature space.^27^

The multilayer perceptron (MLP) is a class of feedforward ANN wherein the biological unit of the brain, the neuron, is modeled by the mathematical unit of a network node.^28^ An MLP consists of at least three layers of nodes: an input layer, one or more “hidden layers,” and an output layer. All nodes except the inputs employ nonlinear activation functions. MLPs use a supervised learning technique called backpropagation to update the parameters of each node. The multilayer structure and nonlinear activations of MLPs distinguish them from linear perceptrons and allow them to categorize data that are not linearly separable. Although MLPs have been successfully applied to practical problems in many fields, these models must be carefully trained and thoughtfully deployed to avoid overfitting or, conversely, failure of convergence during inference.

Convolutional neural networks (CNN) have been widely applied in many tasks.^29^, ^30^, ^31^, ^32^, ^33^, ^34^, ^35^, ^36^ A typical CNN may be composed of several layers performing discrete computational tasks including convolution at various scales of resolution, maximum or other forms of pooling, and batch normalization. The outputs of these layers may be selectively omitted as in dropout or passed as inputs to all subsequent layers when fully connected layers are employed. In order to improve the performance of deep CNNs, various architectures have been proposed. U‐Net adopts symmetrical encoding and decoding paths with skip connections between them and is widely used in medical image segmentation. The residual network (ResNet) architecture employs a shortcut connection which reduces the likelihood of “vanishing” gradients during training, allowing the development of deeper networks.

Supervised learning has been widely used in tumor subregion analysis for the prediction of outcomes including overall survival (OS) or progression‐free survival (PFS), identification of recurrence volume, and subregion segmentation. Sixty‐four papers related to supervised learning are considered in this paper.

#### 4.A.1 Head and neck

CT and ${}^{18}F$‐FDG PET are often used in staging, radiation therapy treatment planning, and evaluation of treatment response in patients with cancers of the head and neck.^37^, ^38^ PET provides detailed functional and metabolic information, while CT reveals the precise anatomical position of the tumor. Table 1 shows a list of selected studies that used supervised learning for tumor subregion analysis in the head and neck. Ding et al. investigated the clinicopathological characteristics of different supraglottic subregions and their correlation with the prognosis of patients with squamous cell carcinoma.^39^ Supraglottic squamous cell carcinomas were divided into four types based on subregion: epiglottis, ventricular bands, aryepiglottic fold, and ventricle. A Cox proportional hazards model was used to generate a biomarker. They found that there were significant differences in the regional control rate, overall survival rate, and cancer‐specific survival rates among different subregions, indicating that patients with carcinoma of the epiglottis or ventricular bands had an increased survival rate relative to those with the disease in the aryepiglottic fold or ventricle. Radiomics is a quantitative method to extract medical image features, such as shape, and texture, that captures tumor heterogeneity.^40^, ^41^, ^42^ Beaumont et al.^43^ developed a voxel‐wise ML radiomics model to identify subregions with tumor recurrence and to predict their location based on pretreatment PET images. An RF model was trained with voxel‐wise features. Voxel‐wise analysis based on radiomic features and spatial location within the tumor was shown helpful in determining the location of recurrence and providing guidance to tailor chemoradiation therapy (CRT) through dose escalation within the area of radiation resistance.

**Table 1:** Overview of supervised learning for tumor subregions analysis based on medical imaging for HN.

Reference	Year	Model	Task	Modality	# of patients in training/testing datasets	Validation method
^39^	2017	Cox proportional hazards model	Predict OS	MRI, CT	111 (N/A)	*P* value
^43^	2019	RF	Recurrence volume identification	${}^{18}F$‐FDG PET/CT	26, LOOCV	AUC

Abbreviations: indicating that the paper only provides the total number of samples; LOOCV, leave‐one‐out cross‐validation; N/A, not available.

#### 4.A.2 Gliomas

Gliomas are the most common primary brain tumor and can be classified into two groups by histopathologic features: high‐grade gliomas (HGG) and low‐grade gliomas (LGG). Magnetic resonance imaging (MRI) provides high soft tissue contrast and is the primary imaging modality to noninvasively diagnose brain tumors.^44^ Dividing gliomas into substructures played an important role in glioma diagnosis, staging, monitoring, and treatment planning for patients. Table 2 shows a list of selected studies using supervised learning in tumor subregion analysis in glioma.

**Table 2:** Overview of supervised learning for tumor subregions analysis based on medical imaging for gliomas.

Reference	Year	Models	Task	Modality	# of patients in training/testing datasets	Validation method
^45^	2018	LDA, QDA, SVM	Predict active and infiltrative tumorous subregions	T1W, T2W, FLAIR, T2‐relaxometry, DWI, DTI, IVIM, and DS‐MRI	10, LOOCV	AUC
^46^	2017	SVM, KNN, Naïve Bayes	Predict overall survival	T1W‐ce, FLAIR, T2W	79, LOOCV	Accuracy
^47^	2019	logistic regression	Identify specific subregions for targeted therapy	DTI	115, (N/A^a^)	*P* values from log‐rank test.
^48^	2017	CNN, LASSO	Predict OS	T1W, T1‐Gd, FLAIR, T2W	75/37	C‐index
^49^	2020	DeepMedic, SVM	Predict PFS and RP	T1W, T1‐Gd, FLAIR, T2W, DWI, DS‐MRI	Scheme 1 and 3:80, 10 fold, Scheme 2: 56/24	AUC
^50^	2018	RF	Predict isocitrate dehydrogenase 1 genes (IDH1)	T1W, T1‐Gd, FLAIR, T2W	118/107	AUC, F1‐score, and accuracy
^51^	2018	RF	Predict survival time	T1W‐ce, FLAIR	73, LOOCV	AUC
^52^	2018	RF	Predict OS and PFS	T1W, FLAIR	40, 5 folds	AUC
^53^	2019	SVM	Glioma grading	DTI, T1W‐ce, FLAIR, T2W‐FSE, DSCE‐RAW, 1H‐MRS	40, LOOCV	Sensitivity, specificity, accuracy, and AUC
^54^	2019	LASSO	stratify glioblastoma patients based on survival	T1W, T1W‐CE, FLAIR, T2W	70/35	C‐index
^55^	2019	Cox proportional hazards model	stratify glioblastoma patients based on survival	post‐T1W	85/42	AUC
^56^	2018	CNN	Tumor subregions segmentation	T1W‐CE	186/47	DSC

Abbreviations: CNN, convolutional neural networks; KNN, k‐nearest neighbors; LASSO, least‐absolute‐shrinkage‐and‐selection‐operator; LDA, linear discriminant analysis; QDA, quadratic discriminant analysis; RF, random forest; SVM, support vector machine.

^a^ Exact training and testing datasets are not available.

Kazerooni et al.^45^ developed a model to discriminate glioma tissue subregions based on multiparametric MRI (mpMRI). Based on histopathologic results, subregions were categorized into active tumor (AT), infiltrative edema (IE), and normal tissue (NT). Fischer's linear discriminant analysis (LDA), quadratic discriminant analysis (QDA), and SVM^57^ were applied to distinguish the three tissue subtypes from each other based on selected features derived from subregions. All three classifiers achieved high classification performance (AUC ~ 90%) with a combination of the following features: characterization of active (CBV), mean diffusivity (MD), high‐resolution T2w image ($T_{2}$_ISO), fluid‐attenuated inversion recovery image (FLAIR). This approach might be advantageously employed to locate tissue subregions prior to image‐guided biopsy procedures. Some studies further predicted OS or PFS based on tumor subregion analysis.^46^, ^47^

Zhou et al.^46^ developed a framework to identify tumor subregions based on pretreatment MRI for patients with glioblastoma (GBM), correlating the image‐based spatial characteristics of subregions with survival time Two datasets were included in this study. Habitat‐based features were extracted from GBM subregions derived from intratumoral grouping and spatial mapping. The results revealed that habitat‐based features were effective for predicting two survival groups with great accuracy (87.5% and 86.36%, respectively). These two survival groups include 32 and 22 GBM patients who did not undergon resection, respectively. The results generated by classifiers (SVM, k‐nearest neighbors (KNN), and naϊve Bayes) showed that the spatial correlation features between the signal‐enhanced subregions can effectively predict survival group (*P* < 0.05 for all classifiers). GBM is further characterized by infiltrative growth at the cellular level that cannot be completely resected. Diffusion tensor imaging (DTI) has been shown to potentially detect tumor infiltration by reflecting microstructural destruction. To investigate the incremental prognostic value of infiltrative patterns over clinical factors and identify specific subregions that may be suitable for targeted therapy, Li et al.^47^ explored the heterogeneity of GBM infiltration using joint histogram analysis in DTI. The prognostic value of covariates for OS and PFS at 12 and 18 months were tested using a logistic regression model. The results showed that joint histogram features have incremental prognostic value when combined with clinical factors, suggesting that patients may benefit from adaptive radiation therapy strategies based on prognostic data obtained during and after treatment if these high‐risk tumor subregions can be identified.

CNNs have achieved tremendous success in tumor subregion analysis and can be used to extract features and segment tumor subregions. Small sample size is one problem encountered with the application of CNNs to limited medical images. Transfer learning and fine‐tuning may be employed to ameliorate small sample problems, making CNNs more useful in medical image tasks.^58^ Lao et al. extracted features from manually segmented tumor subregions based on multimodality MR images and used these features to generate a proposed signature based on LASSO.^48^ The extracted features were of two types: hand‐crafted features and those extracted by a pretrained DL model. The study demonstrated features extracted by pretrained deep learning (e.g., transfer learning) were able to generate imaging signatures for OS prediction and risk stratification for GBM, indicating the potential of DL feature‐based biomarkers in the preoperative care of patients with GBM. CNNs can also be used to segment tumor subregions to facilitate their further study. Based on multiparametric MRI, Kazerooni et al.^49^ constructed a multiinstitutional radomics model that supports up‐front prediction of PFS and recurrence pattern (RP) in patients diagnosed with GBM at the time of initial diagnosis. The proposed framework included subregion identification (DeepMedic^59^), feature extraction, sequential forward feature selection, biomarker generation, and classification. All steps were completed by using the Cancer Imaging Phenomics Toolkit (CaPTk) open‐source software. The area under the receiver operating characteristic curve (AUC) for PFS prediction was 0.88 and 0.82–0.83; AUC for RP was 0.88 and 0.56–0.71 for the single‐institution and multiinstitutional analyses, respectively. The results suggest that the biomarkers included in the radiomics models as implemented in CaPTK could predict PFS and RP in patients diagnosed with GBM. Isocitrate dehydrogenase 1 (IDH1) is established as a prognostic and predictive biomarker for patients with GBM.^60^, ^61^, ^62^, ^63^, ^64^, ^65^ Li et al.^50^ developed a model to predict IDH mutation status in GBM preoperatively based on multiregion radiomic features derived from mpMRI. The proposed model was tested on an independent validation cohort. IDH1 mutation was predicted by the RF model after using Boruta^66^ for feature selection. The multitumor subregions were automatically segmented using a CNN.^67^ The model achieved 97% accuracy with AUC 0.96, and an F1 score of 0.84. The multiregion model built using all‐region features performed better than single‐region models. The multiregion model achieved the best performance when combining age with all‐region features. The results showed that the proposed model based on multiregional mpMRI features has the potential to detect IDH1 mutation status in GBM patients prior to surgery.

#### 4.A.3 BraTS challenge

Glioma subregion segmentation may play an important role in future glioma diagnosis, staging, and treatment planning. Most of the research described here uses nonpublic or institutional datasets, making it difficult to compare methods or results against other published work. The BraTS challenge stands in contrast to these, providing preoperative mpMRI scans sourced from multiple institutions to evaluate the reproducibility of state‐of‐the‐art methods for glioma brain tumor segmentation.^68^, ^69^, ^70^, ^71^, ^72^ The dataset includes images of four MR sequences: T1, T1‐Gd, T2, and FLAIR and four class labels (0: healthy tissues, 1: necrotic core, 2: edema, 3: nonenhancing core 4: enhancing tumor). The evaluation system divides the tumor into three regions for performance evaluation according to practical clinical application: (1) the whole tumor region with labels 1, 2, 3, and 4; (2) the tumor core with labels 1, 3 and 4; (3) the enhancing tumor region (label 4). The nonenhancing core label (label 3) has been eliminated and combined with necrotic core (label) since BraTS 2017.

Table 3 contains a list of selected references using supervised learning methods in tumor subregion analysis of BraTS challenge data. Most are based on DL with various architectures and attention gates are commonly adopted to improve performance by automatically highlighting informative elements of intermediate feature maps.^73^ Hu et al. proposed a novel 3D refinement module that can aggregate local detail information and 3D semantic context directly within the 3D convolutional layer.^74^ Kamnitsas et al. developed a 3D‐CNN with a dual pathway and 11 convolutional layers.^59^ In order to cope with the computational burden of the 3D network, the processing of adjacent image paths was combined into a single channel through the network during training, while automatically adapting to the inherent class imbalances existing in the data. A dual‐path architecture was used to simultaneously process multiscale input images to obtain multiscale context information. A 3D fully conditional random field (CRF) was used in postprocessing and proved effective in mitigating false positives. Havaei et al. developed a novel CNN with a two‐pathway architecture which simultaneously extracted both local and global contextual features^75^. They modeled local label dependencies by cascade‐CNN rather than CRF. This method improved computational speed by using convolution operations rather than CRFs. The success of attention mechanisms in computer vision generally^76^, ^77^, ^78^, ^79^, ^80^, ^81^ and in medical image analysis in particular^73^, ^82^, ^83^, ^84^, ^85^, ^86^ prompted Zhang et al. to integrate an attention gate into a U‐Net architecture, generating an Attention Gate Residual U‐Net (AGResU‐Net) model for brain tumor segmentation.^87^ Several attention gate units were added to the skip connection of the U‐Net to highlight contrast information while minimizing irrelevant and noisy feature responses.

**Table 3:** Overview of supervised learning in tumor subregion analysis of BraTS challenge data.

**Reference**	**Year**	**Models**	**Task**	**# of patients in training/testing datasets**
^75^	2017	Cascade CNN	Tumor subregion segmentation	60, 7 fold
^59^	2017	Efficient Multi‐scale U‐Net with CRFs	Tumor subregion segmentation	253, 5 fold
^74^	2020	3D refinement U‐Net	Tumor subregion segmentation	274/110
^87^	2020	Attention Gate ResU‐Net	Tumor subregion segmentation	285/46, 285/66, 335/125
^88^	2018	Ensemble CNN	Tumor subregion segmnetation	285 (N/A)
^89^	2019	multi‐cascaded CNN with CRFs	Tumor subregion segmentation	40, 274, 285
^90^	2019	*3*D dilated multifiber U‐Net	Tumor subregion segmentation	285/66
^91^	2020	Cross‐task Guided Attention U‐Net	Tumor subregion segmentation	274/110, 285/46, 285/66
^92^	2019	2D‐3D context U‐Net	Tumor subregion segmentation	235/50/46
^93^	2018	CNN	Tumor subregion segmentation	240/34
^94^	2019	Inception‐based U‐Net	Tumor subregion segmentation	165/55/54, 171/57/57
^95^	2018	FCNN with CRFs	Tumor subregion segmentation	30/35, 274/110, 274/191
^96^	2018	SVM, RF, Logistic regression	Glioma grading	285, 5 fold
^97^	2020	U‐Net, RF	Tumor subregion segmentation Predict OS	268/67, 76/29
^98^	2019	LASSO	Predict OS	163, 5 fold
^99^	2020	Heterogeneous CNN with CRFs‐ Recurrent Regression	Tumor subregion segmentation	60 (N/A)
^100^	2019	2.5D cascade CNN	Tumor subregion segmentation	285/46/146, 285/66/191
^101^	2020	IOU 3D symmetric fully CNN	Tumor subregion segmentation	134/33
^102^	2020	CNN	Tumor subregion segmentation	20/10, 192/82, 285/146, 285/191
^103^	2020	CNN, SVM	Tumor subregion segmentation	274, 10 fold
^104^	2018	CNN	Tumor subregion segmentation	274/110
^105^	2019	CNN	Tumor subregion segmentation	285/46, 285/66
^106^	2020	U‐Net	Tumor subregion segmentation	285/46, 285/66
^107^	2018	Hybrid pyramid U‐Net	Tumor subregion segmentation	285, 5 fold
^108^	2019	CNN	Tumor subregion segmentation	285 (N/A)
^109^	2020	CNN	Tumor subregion segmentation	27/254,285
^110^	2020	CNN	Tumor subregion segmentation	85/200
^111^	2020	CNN	Tumor subregion segmentation	68/8, 50/6

Abbreviations: CNN, convolutional neural networks; CRF, conditional random field; N/A, exact training and testing datasets are not available; RF, random forest; SVM, support vector machine.

Table 4 lists the three top‐performing studies from 2017 to 2019 with their results. Ensemble learning, cascade learning, and multiscale operations are commonly added to CNNs to improve the accuracy of brain tumor subregion segmentation. In statistics and machine learning, ensemble learning combines models to surpass the performance of anyone constituent model and is commonly used to improve classification, prediction, and segmentation performance. Kamnitsas et al.^112^ developed an ensemble of multiple models and architectures (EMMA) which combines several DL models for robust segmentation. EMMA independently trained constituent DeepMedic,^59^ FCN,^113^ and U‐Net^114^ models, combining their segmentation predictions at test time. Myronenko et al. proposed a semantic segmentation CNN with asymmetric large encoders to segment tumor subregions.^115^ A variational autoencoder (VAE) branch was added to the network to reconstruct the input images jointly with the segmentation and regularize the shared encoder. Finally, they assembled 10 models trained from scratch to further improve performance. Zhao et al.^116^ developed a self‐ensemble U‐Net, combining multiscale prediction to boost accuracy with a slight increase in memory consumption. They also used the average of all models in the final ensemble and averaged the prediction of overlapping patches to obtain a more accurate result. Cascade learning is a particular case of ensemble learning based on the concatenation‐in‐series of several models, using preceding model outputs as inputs for the next model in the cascade. Wang et al. trained three networks for cascade learning, each with a similar structure, including a large encoder with dilated convolutions and a basic decoder.^117^ The whole tumor was segmented first and a bounding box for the result was used to localize the tumor core. The enhancing tumor was then localized and segmented using the bounding box surrounding the tumor core. The 3 × 3 × 3 convolutional kernel was decomposed into 3 × 3 × 1 and 1 × 1 × 3 kernels to reduce the number of parameters and cope with anisotropic receptive fields. Jiang et al.^118^ developed a two‐stage cascaded U‐Net to segment brain tumor subregions from coarse to fine‐scale. In the first stage, a U‐Net predicts a coarse segmentation result based on the multimodal MRI. The coarse segmentation provides the rough locations of tumors and is used to highlight contrast information. The coarse segmentation results are combined with raw input images prior to input into a second U‐Net with two decoder paths (one using a deconvolution, the other using trilinear interpolation) to generate a fine segmentation map. Zhou et al.^85^ proposed an ensemble framework combining different networks to segment tumor subregions with more robust results. The framework considered multiscale information by segmenting three tumor subregions in cascade with a shared backbone weight and an attention block. Multiscale and deeper networks may achieve better segmentation results because brain tumors have a highly heterogeneous appearance on MR images. Mckinly et al.^119^ proposed a U‐Net‐like network containing a DenseNet with dilated convolutions which also introduced a new loss function, a generalization of binary cross‐entropy, to solve label uncertainty. In another study, Mckinly et al.^120^ used a similar structure but replaced batch normalization with instance normalization and added a simple local attention mechanism between dilated dense blocks. This study included more data for training to further improve network performance. Isensee et al. made minor modifications to U‐Net, replacing ReLU and batch normalization with leaky ReLU and instance normalization to achieve competitive performance.^121^ They also supplemented with data from their own institution to achieve a 2% increase in Dice similarity coefficient (DSC) on the enhancing tumor training data.

**Table 4:** Overview of the top 3 segmentation performance of the last three BraTS (2017–2019).

Reference	Year	Ranking	DSC			HD95 (mm)		
			WT	TC	ET	WT	TC	ET
^112^	2017	1	0.886	0.785	0.729	5.01	23.10	36.00
^117^	2017	2	0.874	0.775	0.783	6.55	27.05	15.90
^122^	2017	3	0.858	0.775	0.647	N/A	N/A	N/A
^123^	2017	3	N/A	N/A	N/A	N/A	N/A	N/A
^115^	2018	1	0.884	0.815	0.766	3.77	4.81	3.77
^121^	2018	2	0.878	0.806	0.779	6.03	5.08	2.90
^119^	2018	3	0.886	0.799	0.732	5.52	5.53	3.48
^85^	2018	3	0.884	0.796	0.778	5.47	6.88	2.94
^118^	2019	1	0.888	0.837	0.833	4.62	4.13	2.65
^116^	2019	2	0.883	0.861	0.810	4.80	4.21	2.45
^90^	2019	3	0.890	0.830	0.810	4.85	3.99	2.74

Abbreviations: ET, enhancing tumor; HD95, Hausdorff distance (95%); N/A, not available; TC, tumor core; WT, whole tumor.

In the past 3 yr, BraTS has also focused on the prediction of OS. Table 5 lists the top three results. RF regression was a popular method for this task. Shboul et al.^124^ extracted 1366 textures and other features, selecting significant features in three steps. The 40 most significant features were used to train the RF regression model and predict OS. Puybareau et al.^125^ extracted features from the segmented tumor region and introduced patient age into the feature space. Principal component analysis (PCA) was performed to normalize the training set. The feature‐wise mean, standard deviation, and projection matrix (W) were computed and stored during the rescaling phase of the PCA. The RF regression model was trained based on the normalized data. The feature vector of the test set was also normalized by the feature‐wise mean and standard deviation derived from the training phase, and was then projected in the principal component space with W. The rescaled vectors were fed into the trained RF classifiers and the final prediction was obtained by majority voting. Sun et al.^126^ extracted 4526 tumor features based on prior segmentation results. Important features were selected by decision tree and cross‐validation. Finally, they trained an RF regression model to predict OS. MLP was another popular method for this task. Jungo et al.^127^ computed 26 geometrical tumor features and added age to complete the feature space. The four most important features were selected before being fed into a fully connected neural network with one hidden layer and a linear activation function. Baid et al.^128^ extracted tumor features and excluded high‐correlation features by Spearman testing. An MLP was trained using variables correlated with OS. Wang et al.^129^ selected seven features as input for a fully connected neural network with two hidden layers. Their linear regression model also achieved good results. Feng et al.^130^ used imaging features and clinical variables in a linear regression model. They used two‐dimensional feature vectors to represent clinical resection status and compensate for sparse data. They used a linear regression model to fit the training data after feature normalization. Weninger et al.^131^ measured the volume of subregions based on segmentation results. The volume information, the distance between centroids representing tumor and healthy brain, and patient age were used as input to linear regression for prediction of OS. In addition to radiomic features, Wang et al.^132^ also considered biophysical modeling of tumor growth and calculated the ratio of second semi‐axis length between tumor core and whole tumor to define a novel relative invasiveness coefficient (RIC). Following feature selection, RIC, age and radomic features were fed into the epsilon‐support vector regression. The method achieved an accuracy of 0.56 in OS prediction by incorporating RIC.

**Table 5:** Overview of the studies and results with top 3 OS prediction performance of each year from 2017 to 2019.

**Reference**	**Year**	**Ranking**	**Accuracy**	**MSE**	**Median‐SE**	**Std‐SE**
^124^	2017	1	0.579	245779.5	24944.4	726624.7
^127^	2017	2	0.568	213000.0	28100.0	662600.0
^133^	2017	3	N/A	N/A	N/A	N/A
^130^	2018	1	0.612	231746.0	34306.4	N/A
^125^	2018	2	0.605	N/A	N/A	N/A
^126^	2018	2	0.605	N/A	32895.1	N/A
^128^	2018	3	0.558	338219.4	38408.2	939986.8
^131^	2018	3	0.558	277890.0	43264	N/A
^134^	2019	1	0.579	374998.8	46483.36	1160428.9
^132^	2019	2	0.56	N/A	N/A	N/A
^135^	2019	3	0.551	N/A	N/A	N/A
^129^	2019	3	0.551	41000.0	49300.0	123000.0

Abbreviations: MSE, mean square error; N/A, not available.

### 4.B Unsupervised learning in tumor subregion analysis of medical images

Supervised learning requires time‐consuming and labor‐intensive manual data annotation. In contrast, unsupervised techniques learn the distribution of input data and divide samples into clusters without a labeled training dataset. Unsupervised learning has been widely used in tumor subregion analysis. Of the twenty‐four papers employing unsupervised techniques in Table 6, most focus prediction on OS, PFS, or identification of tumor recurrence. Common unsupervised learning algorithms include level set methods (LSM), thresholding, active contour modeling (ACM), hidden Markov random fields (HMRF), the K‐means, and expectation‐maximization (EM) algorithms, principal component analysis (PCA), individual‐ and population‐level clustering and hybrid hierarchical clustering. ACM works to segment objects in an image by evolving a curve according to the constraints in the image.^136^ The HMRF model is a random process generated by MRF. Its state sequence cannot be directly observed, but can be indirectly estimated through observation.^137^ The EM algorithm is an iterative method that searches the (local) maximum likelihood or maximum a posteriori (MAP) estimate of the parameters in a statistical model.^138^ PCA is an orthogonal linear transformation that reduces the dimensionality of the input data while retaining its most significant parts.^139^ K‐means identifies k centroids and assigns each data point to the nearest centroid by minimizing the sum of the squared Euclidean distances between each point and its assigned centroid.^140^ Hybrid hierarchical clustering combines the advantages of bottom‐up hierarchical clustering and top‐down clustering, so it is applicable to large and small datasets alike.^141^ Fuzzy C‐means clustering (FCM) is a data clustering method which allows each data to belong to each cluster to a certain degree.^142^ Global‐initiated regularized local fuzzy clustering (GRELFC) is a method first globally initiates training to identify fuzzy clustering of physiological imaging parameters in the feature space, and then classifies each tumor volume into sub‐volumes by local regularization based on global feature clustering.^143^ GRELFC choose FCM as the fuzzy clustering method. Fuzzy local adaptive Bayesian (FLAB) is an unsupervised statistical method based on the Bayesian framework^144^

**Table 6:** Unsupervised learning for tumor subregions analysis.

Reference	Year	Models	Modality	Task	ROI	# of patients in training/testing datasets
^145^	2016	Level set, MRF, EM	Post‐T1W, FLAIR	Predict OS	Brain	46/33
^146^	2017	level set	T1W‐ce, DWI	Predict OS	Brain	62/46
^147^	2019	Threshold, Cox proportional hazards	(11)C‐MET‐PET, T1W‐Gd, FLAIR	Recurrence tumor identification, predict PFS	Brain	37 (N/A)
^148^	2014	Threshold, SVM, Naïve Bayes, decision tree, wrapper, CFS	DCE‐MRI	Estrogen receptor (ER) classification	Chest	20, LOOCV
^149^	2016	Individual‐ and population‐level clustering	${}^{18}F$‐FDG PET/CT	Predict OS and OFD	Chest	44 (N/A)
^150^	2020	Individual‐ and population‐level clustering	${}^{18}F$‐FDG PET/CT	Assess early response and predict PFS	HN	162, 10 fold
^151^	2018	Individual‐ and population‐level clustering	DCE‐MRI	Predict RFS	Chest	60/186
^152^	2019	Individual‐ and population‐level clustering LASSO	${}^{18}F$‐FDG PET/CT	Predict PFS	HN	85/43
^153^	2017	Individual‐ and population‐level clustering	PDG PET, CT, DCE‐MRI, HX4 PET	Predict OS	Chest	36 (N/A)
^154^	2019	K‐means, LASSO	CT	Predict OS	HN	87/46
^155^	2016	k‐means	DCE‐MRI	Recurrence tumor identification	Pelvis	81 (N/A)
^156^	2020	K‐means, PCA	DCE‐MRI	Tumor subregion segmentation	Abdomen	14 (N/A)
^157^	2016	ACM	Post‐T1W, FLAIR, T2W	Tumor subregion segmentation	Brain	4 (N/A)
^13^	2018	K‐means	DCE‐MRI	Predict prognosis	Chest	77, LOCCV
^158^	2019	FCM, CAM	DCE‐MRI	Predict OS and RFS	Chest	61/173/87
^159^	2014	GIRLFC	DCE‐MRI	Predict tumor progression after RT	Abdomen	20 (N/A)
^160^	2019	FLAB	${}^{18}F$‐FDG PET/CT	Tumor subregion segmentation	HN	54 (N/A)
^143^	2012	GIRLFC	DCE‐MRI	Predict subvolume related to treatment outcome	HN	14 (N/A)
^161^	2019	3D Level set	${}^{18}F$‐FDG PET/CT	Predict OS	Chest	30 (N/A)
^162^	2018	PCA	DCE‐MRI, DWI, PET/CT	Predict neoadjuvant therapy response	Chest	35 (N/A)
^163^	2019	CAM, RF	DCE‐MRI	Predict breast cancer subtypes	Chest	211. LOOCV
^164^	2020	TTP, SVM, LASSO	DCE‐MRI	Predict HER2 2+ status in breast cancer	Chest	76, LOOCV
^165^	2020	FLAB	18F‐FDGPET/CT	Recurrence tumor identification	Plevis	21 (N/A)
^166^	2019	K‐means	DWI, PET	Segmentation and Predict PFS	Chest	18, LOOCV

Abbreviations: CAM, convex analysis of mixtures; FCM, fuzzy C‐means; FLAB, fuzzy locally adaptive Bayesian; GIRLFC, global‐initiated regularized local fuzzy clustering; HN, head and neck; LASSO, least‐absolute‐shrinkage‐and‐selection‐operator; PCA, principal component analysis; RF, random forest; SVM, support vector machine; TTP, time to peak.

#### 4.B.1 Level set methods

Level set methods are commonly used for unsupervised segmentation tasks. Cui et al.^145^ developed and validated prognostic imaging biomarkers to predict OS in GBM based on multiregion quantitative image analysis. Each tumor was semi‐automatically segmented by the level set algorithm and then further divided into subregions using the hidden Markov random field (MRF) model and EM algorithm.^137^ The biomarker was generated based on LASSO to predict the OS, and the model was tested on an independent institutional cohort. The concordance index and stratification of OS using the log‐rank test were 0.78 and *P* = 0.018 for the proposed method, outperforming conventional prognostic biomarkers such as age (concordance index: 0.57, *P* = 0.389) and tumor volume (concordance index: 0.59, *P* = 0.409). In a later study, Cui et al.^146^ defined a high‐risk volume (HRV) based on mpMRI images for predicting GBM survival and investigated its relationship and synergy with molecular characteristics. Each tumor was delineated by the level set algorithm. The manual correction was performed for eight failed cases. The patients with an unmethylated MGMT promoter and high HRV had significantly shorter OS (median 9.3 vs 18.4 months, log‐rank; *P* = 0.002), indicating the volume of the high‐risk tumor subregion identified on mpMRI can predict survival and complement genomic information.

#### 4.B.2 Threshold‐based methods

Threshold algorithms are also suitable to separate tumor subregions based on imaging characteristics. Miller et al.^147^ investigated whether three month treatment response of newly diagnosed GBM based on C‐methionine‐positron emission tomography (MET‐PET) could predict prognosis better than baseline MET‐PET or anatomic magnetic resonance imaging alone. A threshold set at 150% of mean cerebellar uptake was used to automatically segment the metabolic tumor volume (MTV). Persistent MTV at three months was defined as the overlap of the three month MTV and the pre‐treatment MTV. Cox proportional hazards were used in multivariate analysis of PFS and OS. Results showed that most patients (67%) with gross total resection (GTR) of newly diagnosed GBM have measurable postoperative MTV and that the total and persistent MTV three months post‐CRT were predictors of PFS. GTV‐Gd at recurrence encompassed 97% of the persistent MET‐PET subvolume, 71% of the baseline MTV, 54% of the baseline GTV‐Gd, and 78% of the three month MTV. Persistent MET‐PET subvolume best predicts the location of tumor recurrence.

Estrogen receptor (ER) status is a recognized molecular feature of breast cancer correlated with prognosis and its early detection can significantly improve treatment efficacy by guiding selection of targeted therapies.^167^ Chaudhury et al. developed a novel framework to classify ER status by extracting textural kinetic features from peripheral and core tumor subregions.^148^ The whole tumor was segmented using automatic threshold selection^168^ combined with morphological dilation and connected component analysis. The whole tumor was divided into two subregions according to tumor geometry. Two feature selection methods (wrapper^169^ and correlation‐based feature subset selection (CFS)^170^) and three classifiers (naϊve Bayes,^171^ SVM,^27^, ^172^ decision tree^173^) were adopted in this study and each feature selector followed a classifier, for a total of six model composition combinations. The best classification accuracy approached 94%, indicating that subregion texture feature extraction can accurately classify ER status.

#### 4.B.3 Individual‐ and population‐level clustering

Individual‐ and population‐level clustering are used to assign each pixel or voxel to suitable clusters in order to divide a tumor into subregions. After tumor subregions are obtained, the relationship between tumor subregions, OS and PFS can be investigated.

Wu et al. used individual‐ and population‐level clustering in three works related to tumor subregion analysis. In one of their studies,^149^ they developed a robust tumor partitioning method to identify high‐risk subregions in lung cancer. The method divided the tumor using a two stage process: each tumor was first divided into homogeneous subregions (i.e., super pixels) at the patient‐level on PET and CT images via K‐means clustering. ^140^ These superpixels were then merged into subregions via population‐level hierarchical clustering.^174^ High‐risk subregions predicted OS and out‐of‐field progression (OFP) over the entire cohort with a C‐index of 0.66–0.67. For patients with stage III disease, the C‐index reached 0.75 (HR 3.93, log‐rank *P* < 0.002) and 0.76 (HR 4.84, log‐rank *P* < 0.002) for predicting OS and OFP, respectively. In contrast, the C‐index was lower than 0.60 for traditional imaging markers. The volume of the most metabolically active and heterogeneous solid components of the tumor predicted OS and OFP better than conventional imaging markers. In a second study, Wu et al.^150^ developed an imaging biomarker to assess early treatment response and predicted outcomes in oropharyngeal squamous cell carcinoma (OPSCC). Based on ${}^{18}F$‐FDG PET and contrast CT imaging, the primary tumor and involved lymph nodes were divided into subregions by individual‐ and population‐level clustering. The proposed imaging biomarker was generated by the LASSO algorithm. The C‐index was 0.72 for the training set and 0.66 for the validation set, suggesting the proposed biomarker can accurately predict disease progression and provide patients with better risk‐adapted treatment. In a third study investigating risk‐stratification in breast cancer, Wu et al. divided each tumor into spatially segregated, phenotypically consistent subregions based on individual‐ and population‐level clustering, and used a net strategy to construct an imaging biomarker based on image features derived from the multiregional spatial interaction (MSI) matrix.^151^ This revealed three intratumoral subregions with distinct perfusion characteristics, with results suggesting tumor heterogeneity may be an independent predictor of recurrence‐free survival (RFS), independent of traditional predictors.

In order to predict PFS in patients with nasopharyngeal carcinoma (NPC), Xu et al. extracted subregion features via individual‐ and population‐level clustering to generate a biomarker by LASSO.^152^ Three subregions ($S_{1}$, $S_{2}$, $S_{3}$) with distinct PET/CT imaging characteristics were obtained. The C‐index and log‐rank test for imaging biomarker $S_{3}$ and whole tumor are 0.69 and 0.58, and *P* < 0.001 and *P* < 0.552, respectively, indicating $S_{3}$ is superior to whole tumor in terms of prognostic performance. Imaging biomarker $S_{3}$ and American Joint Committee on Cancer (AJCC) stages III–IV were identified as independent predictors of PFS based on multivariate analysis (P=0.011 and P=0.042, respectively). When combined to form a scoring system, imaging biomarker $S_{3}$ and AJCC stages III‐IV outperformed AJCC staging alone (log‐rank test *P* < 0.0001 vs 0.0002; *P* < 0.0021 vs 0.0277 for the primary and validation cohorts, respectively). The results demonstrated that PET/CT subregion radiomics was able to predict PFS in NPC and provide prognostic information to complement other established predictors.

Even et al.^153^ designed a subregion analysis for nonsmall cell lung cancer (NSCLC) using multiparametric imaging. The multiparametric images were divided into subregions in two steps: each tumor was first divided into homogeneous subregions (i.e., super voxels) before being segregated into phenotypic groups by hybrid hierarchical clustering.^141^ Patients were clustered according to the absolute or relative volume of super voxels. The results showed that hypoxia, FDG avidity, and an intermediate level of blood flow/volume indicated a high‐risk tumor type with poorer survival (P=0.035), providing evidence of the prognostic utility of subregion classification based on multiparametric imaging in NSCLC.

#### 4.B.4 K‐means

K‐means is a popular unsupervised learning method that partitions samples into k clusters. Xie et al. developed a survival prediction model for patients with esophageal squamous cell carcinoma prior to concurrent CRT.^154^ Tumors were divided into subregions by K‐means clustering. Radiomic features were then extracted from these subregions to construct a biomarker based on the LASSO algorithm and predict OS. Independent patient cohorts from another hospital were used to validate the model. The C‐indices were 0.729 (0.656–0.801, 95% CI) and 0.705 (0.628–0.782, 95% CI) in the training and validation cohorts, respectively. AUC for the 3‐yr survival ROC were 0.811 (0.670–0.952 95% CI) and 0.805 (0.638–0.973, 95% CI), respectively. Such a model may facilitate personalized treatment through accurate prediction of early treatment response.

Torheim et al. used K‐means in MRI imaging of cervical cancer to divide voxels into two clusters based on relative signal increase (RSI) time series. Clusters of hypo‐enhancing voxels demonstrated a significant correlation with locoregional recurrence (P=0.048).^155^ Tumors with poor treatment response exhibited this characteristic in several regions, indicating a potential candidate for targeted radiotherapy.

Franklin et al. developed a method to semi‐automatically segment viable and non‐viable tumor regions in colorectal cancer based on DEC‐MRI and compared these with histological subregions of viable and non‐viable tumor, analyzing extracted pharmacokinetic parameters between them.^156^ The whole tumor was manually delineated and four subregions were automatically obtained by PCA, followed by K‐means. These four subregions were manually merged into two: viable and non‐viable tumors. For viable tumor subregions defined by imaging and histology, DSC = 0.738 indicating the consistency of viable tumor segmentation between pre‐operative DCE‐MRI and postoperative histology. This technique may facilitate non‐invasive assessment of treatment response in clinical practice.

#### 4.B.5 Fuzzy clustering

Fan et al. developed a framework to assess tumor subregion heterogeneity in breast cancer based on the decomposition of DCE‐MR images.^158^ The whole breast tumor was segmented by the FCM algorithm.^175^ A convex analysis of mixtures (CAM) method was then used to differentiate heterogeneous regions. Imaging features extracted from these regions were used to predict prognosis and identify gene signatures. Tumor heterogeneity was negatively correlated with the presence of genetic markers of breast cancer and survival.

Wang et al. studied primary and secondary intrahepatic malignancies to determine whether an increase in tumor subvolume with elevated arterial perfusion during RT can predict tumor progression following treatment.^159^ The arterial perfusion of tumors prior to treatment was clustered into low‐normal and elevated perfusion by GIRLFC.^143^ The tumor subvolumes with elevated arterial perfusion were extracted from the hepatic arterial perfusion images. The changes in tumor sub‐volumes and arterial perfusion averaged over the tumors from pre‐treatment baseline to mid‐treatment were investigated for prediction of tumor progression following treatment. The results showed that an increase in intrahepatic subvolume with elevated arterial perfusion during RT may be a predictor of post‐treatment tumor progression (AUC = 0.9).

Lucia et al. ^165^ developed a framework to evaluate the overlap between the initial high‐uptake sub‐volume ($V_{1}$) on baseline ${}^{18}F$‐FDG PET/CT images and the metabolic relapse ($V_{2}$) after chemoradiotherapy in locally advanced cervical cancer. CT images of recurrence were registered with baseline CT using the 3D Slicer Expert Automated Registration module^176^ to obtain the deformation fields by optimizing the Mattes mutual information metric,^177^ and the corresponding PET images were registered using the corresponding deformation fields. The FLAB algorithm ^144^ was used to determine the sub‐volumes $V_{1}$ and $V_{2}$ for baseline and follow‐up PET images. The overlaps between the baseline high‐uptake sub‐volume and the recurrent metabolic volume were moderate to good (range (mean ± std)): 0.62–0.81 (0.72 ± 0.05), 0.72–1.00 (0.85 ± 0.10), 0.55–1.00 (0.73 ± 0.16) and 0.50–1.00 (0.76 ± 0.12) for DSC, overlap fraction, X $\left(X=\frac{V_{1}\cap V_{2}}{V_{1}}\right)$ and Y$\left(Y=\frac{V_{1}\cap V_{2}}{V_{2}}\right)$, respectively.

#### 4.B.6 Active contour modeling

Seow et al.^157^ segmented the solid subregion of high‐grade gliomas in MRI images by active contour modeling (ACM) and reported a difference ratio (($s_{\mathrm{ACM}}‐S_{\mathrm{manual}}$)/$s_{\mathrm{ACM}}$, where $s_{\mathrm{ACM}}$ and $s_{\mathrm{manual}}$ are ACM and manual segmentation areas, respectively) of 1.3. This algorithm produced segmentations in under 20 min, while manual segmentation required an hour.

## 5 PUBLICLY AVAILABLE TOOLBOXES

There are several publicly available toolboxes that are useful for tumor subregion analysis. Python packages include pyradiomics, used to extract radiomic features,^178^ Scikit‐learn, a general purpose machine learning toolkit^179^; and NiftyNet, a convolutional neural network platform based on TensorFlow for medical image analysis and image‐guided treatment research.^180^ In C++, LIBSVM includes various SVMs, which can be used for classification and regression.^27^

## 6 PREVALENCE OF METHODS

**Fig. 3:**
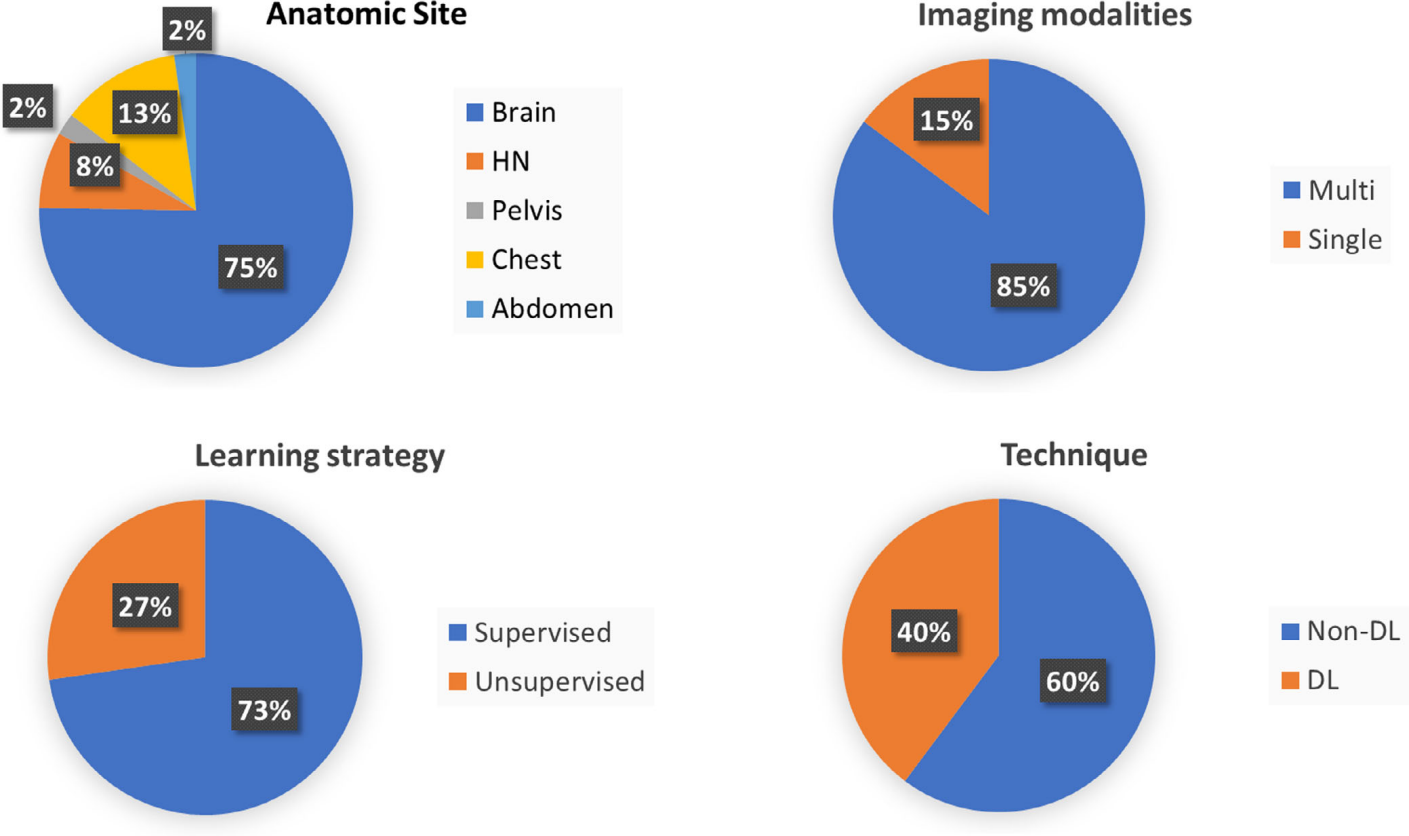
Pie charts for the distribution of various methods in AI‐based tumor subregion analysis in medical imaging. HN, head and neck; DL, deep learning

We have analyzed the prevalence of selected study characteristics, including disease site, learning strategy (supervised/unsupervised), technique (deep learning/others), and imaging modalities (single/multi) (Fig. 3). Brain and thorax are the most studied disease sites, likely secondary to clinical uncertainty in assessing treatment response in the brain and in diagnosis based on thoracic screening imaging. The brain's anatomic position, which is approximately fixed by the surrounding calvarium, facilitates multimodal image registration and is an additional contributor to the brain's popularity as the most common study site in medical imaging. The BraTS challenge further encourages study by providing public data as well as ground‐truth for nonpublic data against which to analyze results. Supervised learning accounts for 73% of works reviewed, owing to the greater reliability and interpretability of training when ground truth is available. Non deep‐learning strategy is employed in 60% of the summarized studies while deep learning strategy accounts for 40%. The category of multimodal studies accounts for 85% of all works while single‐modality studies account for 15%. Magnetic resonance spectroscopy (MRS) plays an important role in detecting visible or invisible metabolic abnormalities^181^ and may be useful for tumor subregion analysis. MRS is adaptable and can be applied to relevant metabolic profiles across different tissues. MRS is currently finding primary use in investigations of the brain but may be used for detection, localization, staging, evaluations of tumor aggressiveness, and response for many cancers such as those of the prostate and breast.

## 7 SUMMARY AND OUTLOOK

AI methods from the field of computer vision have been widely adopted for tasks in tumor subregion analysis. The choice of methods is often based on dataset size and the task at hand. For small sample sizes, support vector machines and random forests are often used for classification and regression problems. Cox proportional hazards models and LASSO are commonly used in regression, and can also aid in feature selection before classification or regression to prevent overfitting. If enough data are available, deep learning approaches are a popular choice for many tasks. As reviewed here, brain is the most commonly studied site followed by thorax. Because brain tumor datasets are generally accompanied by ground‐truth data, supervised learning methods are most commonly employed. For other sites, unsupervised methods are popular due to the lack of ground truth contours of tumor subregions.

Currently, there is no universal image acquisition protocol for subregion analysis in any imaging modality in clinical practice and images acquired from different sites using different protocols may affect the performance of these models. Fave et al. showed that image texture may not be significantly affected by the choice of peak tube voltage of CT, while is affected by a decrease in tube current.^182^ In order to address this issue, the quantitative imaging biomarkers alliance (QIBA)^183^ and the quantitative imaging network (QIN)^184^ have been working to formalize standard imaging protocols.

Sample sizes in the reviewed studies were small to intermediate (median *n* = 230, range *n* = 4‐626). For supervised learning, a large training set is required to train a reliable model. A large validation set is also essential for rigorous evaluation. Except for the BraTS studies, most reviewed here used institutional data and may lack generalizability. Many studies on tumor subregions demonstrate correlations to survival, as well as treatment response and recurrence. To validate these findings, significant time must be invested in follow‐up especially in diseases with low overall mortality. Validation may also be confounded by adjuvant treatment during the follow‐up period, complicating the analysis of any relationships that are discovered. The establishment of universal benchmark datasets would solve many of these problems, providing a standard against which to measure the performance of new techniques.

Deep learning has demonstrated clinical utility in many tasks in medical imaging. At the time of writing, deep learning is primarily in use for brain tumor subregion segmentation but is rarely used in non‐segmentation tasks or in other body sites. Great potential remains for DL applications in tumor subregion analysis. A CNN might be used to automatically extract useful features rather than relying upon handcrafted features, which may be biased by the crafter's prior knowledge of the training data and fail to capture detail that may be observed in test data, or later in inference. Deep features learned from CNNs, on the other hand, maybe more robust to unseen data as they are objectively selected from the feature space through iterative optimization. For clinical tasks for which it is difficult to obtain manually‐annotated ground truth data, unsupervised CNNs have been applied to solve the segmentation problem. Zhou et al. proposed a deep image clustering model to assign pixels to different clusters by updating cluster associations and cluster centers iteratively.^185^ CNN could also be used to generate radiomic signatures for various clinical applications based on tumor subregions and be used in OS prediction, treatment response prediction, and clinical risk stratification. In order to realize the full potential of DL applications in tumor subregion analysis, models must be trained on large datasets with external cross‐site validation. Contrastive learning approaches, wherein traditional cross‐entropy losses are supplanted by maximization of mutual information, have gained popularity in the last year because they effectively utilize unlabeled data for unsupervised learning. Many of these have achieved good results.^186^, ^187^, ^188^ Few‐shot learning comprises two stages: a meta training and a testing stage. In the meta training stage, the data is decomposed into discrete tasks to encourage the generalizability of the model robust to category changes. In the meta testing stage, when facing a new category, classification can occur without changing the existing model. Few‐shot learning may be useful in tumor subregion analysis, a classification problem wherein the sample size is typically small.^14^, ^188^

Although most of the papers included in this review did not report or discuss time for training or testing, this is a critical variable in the real‐world deployment of these technologies as “Big Data” gets bigger: training time may exceed 24 h in the setting of 3D, rather than 2D, tumor segmentation while inference may occur in less than 1 min in production. The development of ever lighter and faster networks offers the opportunity to effectively shorten training time and may outpace hardware‐based innovations.

## AUTHOR CONTRIBUTIONS

Mingquan Lin: Conceptualization, methodology, investigation, writing—original draft, visualization; Jacob F. Wynne: Methodology, writing—review and editing; Boran Zhou: Methodology, writing—review and editing; Tonghe Wang: Writing—review and editing; Yang Lei: Writing—review and editing; Walter J. Curran: Writing—review and editing, supervision; Tian Liu: Writing—review and editing, supervision; Xiaofeng Yang: Conceptualization, methodology, investigation, writing—original draft, supervision, project administration, funding acquisition.
